# Importance of R_2_ accuracy in susceptibility source separation

**DOI:** 10.1002/mrm.70034

**Published:** 2025-08-16

**Authors:** Tereza Beatriz Oliveira Assunção, Nashwan Naji, Jeff Snyder, Peter Seres, Gregg Blevins, Penelope Smyth, Alan H. Wilman

**Affiliations:** ^1^ Department of Biomedical Engineering University of Alberta Edmonton Alberta Canada; ^2^ Department of Radiology and Diagnostic Imaging University of Alberta Edmonton Alberta Canada; ^3^ Division of Neurology, Department of Medicine University of Alberta Edmonton Alberta Canada

**Keywords:** R_2_ map, source separation, susceptibility, transverse relaxation mapping

## Abstract

**Purpose:**

To examine the importance of R_2_ accuracy on independent paramagnetic and diamagnetic outputs from susceptibility source separation in the brain from two publicly available methods.

**Methods:**

The effects of R_2_ errors, which translate into $R_{2}^{\prime }$ errors, on output maps from χ‐separation and χ‐sepnet were examined using data from 11 healthy volunteers. Baseline R_2_ values were determined by Bloch modeling a dual‐echo turbo spin echo decay with measured flip angles. R_2_ errors were introduced from either simple exponential fitting, R_2_ multiplication factors, or R_2_ approximation using only $R_{2}^{*}$. Altered R_2_ maps were then used as input for the susceptibility source separation models using either default or calculated relaxometric constant. Difference maps and mean percentage errors within regions of interest (ROIs) were measured.

**Results:**

Errors in R_2_, and hence $R_{2}^{\prime }$, directly affected paramagnetic and diamagnetic components. χ‐sepnet was less sensitive to R_2_ errors than χ‐separation and had reduced variance among subjects. χ‐sepnet susceptibility component errors did not reach more than ±20% in most ROIs for all alteration approaches. In contrast, χ‐separation, with default relaxometric constant, reached 56% susceptibility component error with −25% R_2_ error input. Exponential fitting R_2_ error exceeded −25%, thus, even larger component errors occurred. $R_{2}^{*}$‐based approximation had −25% R_2_ mean error across ROIs (−18% across whole brain), yielding 57% mean susceptibility component error across ROIs.

**Conclusion:**

Paramagnetic and diamagnetic outputs of susceptibility source separation methods have variable responses to R_2_ error, that may occur with simple R_2_ fitting or R_2_ approximation, and can be strongly biased by it.

## 1 INTRODUCTION

In human brain, the main susceptibility sources are paramagnetic iron and diamagnetic myelin.^1^ Myelin, which forms the insulating sheath around axons, is found throughout brain tissue with higher concentrations in white matter (WM). Iron is an essential element for brain function, including oxygen transport and myelin synthesis. Most of the non‐heme iron in the brain is stored in ferritin proteins. Alterations in iron and myelin content have been shown in demyelinating and neurodegenerative diseases as well as in normal neurodevelopment and aging.^2^, ^3^, ^4^, ^5^

QSM is sensitive to iron and myelin.^6^, ^7^ In areas with high iron concentration, like the basal ganglia, the QSM signal has been interpreted as mainly due to iron, enabling studies of normal and abnormal iron distribution^7^, ^8^, ^9^ and changes in iron concentration across the healthy lifespan.^10^, ^11^ Myelin quantification with QSM is more difficult, but QSM has shown value for identifying myelination in brain development, as well as demyelination and remyelination in multiple sclerosis (MS) lesions.^12^, ^13^, ^14^ Net changes in brain susceptibility are useful for following neurodegenerative diseases, such as, MS,^15^ amyotrophic lateral sclerosis,^16^ Parkinson's disease,^17^ temporal lobe epilepsy^18^ and Alzheimer's disease.^19^ However, QSM cannot quantify subvoxel paramagnetic and diamagnetic changes, since it only provides the net sum. For example, either a myelin decrease or an iron increase could increase the net susceptibility which is especially problematic if iron and myelin changes co‐localize in the same voxel.

Past work has utilized QSM in combination with the effective transverse relaxation rate ($R_{2}^{*}$) to gain more insight into paramagnetic and diamagnetic changes^20^, ^21^; however, these methods did not quantify subvoxel components. A further advance was to take advantage of the known relationship between the static field reversible relaxation rate ($R_{2}^{\prime }$) and susceptibility^22^ to separate the paramagnetic and diamagnetic components based on opposing additive or subtractive effects of $R_{2}^{\prime }$ and susceptibility.^23^ Other works have also proposed similar susceptibility source separation methods, with different processing approaches.^24^, ^25^, ^26^, ^27^, ^28^ These methods have already been applied in the brain for independent analysis of myelin or iron changes in MS, Alzheimer's disease, brain aging and development.^29^, ^30^, ^31^, ^32^, ^33^

$R_{2}^{\prime }$ necessitates the acquisition of both the effective ($R_{2}^{*}$) and the irreversible transverse rate (R_2_); however, the addition of R_2_ can lengthen scan times and is not always acquired in standard susceptibility studies that use only multiple echo gradient echo (MEGE) data. Hence, methods have been introduced to estimate $R_{2}^{\prime }$ or model the source separation without R_2_, thus reducing the additional time requirement.^24^, ^25^, ^26^, ^27^ Although good source separation results have been obtained by approximating R_2_ from $R_{2}^{*}$,^25^, ^27^, ^28^ there are differences from classical $R_{2}^{\prime }$ approaches, that rely on the closer relationship between $R_{2}^{\prime }$ and susceptibility. With the classic $R_{2}^{\prime }$ approach to susceptibility source separation, the accuracy of the $R_{2}^{\prime }$ map (= $R_{2}^{*}$–R_2_) will depend on the accuracy of $R_{2}^{*}$ and R_2_ maps.

While $R_{2}^{*}$ is relatively straightforward to measure from a MEGE sequence, R_2_ measurement is more complex. Common pulse sequences to measure R_2_ include multiple echo spin echo (MESE) or turbo spin echo (TSE) with multiple TEs. Other approaches rely on steady‐state free precession or T_2_ preparation.^34^, ^35^ Using a MESE or TSE approach, the decay curves are strongly affected by the exact refocusing flip angles, which complicates the R_2_ analysis. The RF refocusing flip angles may be altered from 180° by both the transmit B_1_^+^ variation and by the slice profile variation giving rise to substantial stimulated echoes.^36^ In addition, TSE sequences commonly use refocusing flip angles less than 180° to manage RF heating. Accurate modeling of the pulse sequence makes it possible to achieve the correct R_2_, with common methods utilizing the echo phase graph algorithm or full Bloch equations.^37^, ^38^

R_2_ errors may arise from many sources, with the most severe and common arising from simple exponential fitting of the MESE or TSE decay curve. This approach remains common owing to its simplicity without the need for complex modeling of the pulse sequence. However, it is known to underestimate R_2_, even when skipping the first echo in MESE sequences.^39^ In addition, the R_2_ error with exponential fitting of dual‐echo TSE sequences can vastly underestimate R_2_.^40^ Even when modeling the pulse sequence, R_2_ errors might also arise from inaccurate slice profile modeling in 2D sequences or from inaccurate B_1_^+^ approximations when a B_1_^+^ map is not acquired. Another source of R_2_ error for susceptibility source separation would arise when R_2_ mapping data is not collected and only the $R_{2}^{*}$ is available to approximate R_2_ and $R_{2}^{\prime }$.^25^ In general, any errors in R_2_ will translate into errors in $R_{2}^{\prime }$ that may affect the susceptibility source separation outputs. However, the accuracy needed for R_2_ mapping in susceptibility source separation remains unknown.

This work examines the importance of R_2_ accuracy in susceptibility source separation in healthy volunteers using two publicly available pipelines^23^, ^41^ with varying R_2_ inputs achieved through Bloch modeling, simple exponential fitting, global R_2_ scaling, or $R_{2}^{*}$‐based approximation. The results provide an understanding of error tolerance in R_2_ input for mapping diamagnetic and paramagnetic susceptibility components using susceptibility source separation.

## 2 METHODS

### 2.1 Subjects and MRI protocol

Eleven healthy subjects (36 ± 15 y, 7 females), who were part of the local arm of the Canadian Prospective Cohort (CanProCo) study,^42^ were scanned at 3T (Siemens Prisma, Erlangen, Germany) after informed consent. This additional study was approved by the local ethics board. For R_2_ mapping, a 2D dual‐echo TSE (TR = 2500 ms, TE1 = 10 ms, TE2 = 93 ms, turbo factor 8, slice thickness 3.0 mm, 165° refocusing, FOV 240 × 180 × 150 mm^3^, spatial resolution 0.9 × 0.9 × 3.0 mm^3^, scan time 3 min 9 s) was used in addition to a Bloch‐Siegert B_1_^+^ mapping sequence^43^ (spatial resolution 1.3 × 1.3 × 3.0 mm^3^, scan time 39 s). The B_1_^+^ map enables determination of the actual flip angles in each voxel which is required for the pulse sequence modeling. For $R_{2}^{*}$ mapping, phase and QSM, a 3D MEGE (TR = 47 ms, TE1 = 5.0 ms with echo spacing 7.1 ms, 6 echoes, flip angle 18°, slice thickness 2.0 mm, FOV 240 × 187 × 144 mm^3^, 0.6 × 0.7 × 2.0 mm^3^, scan time 6 min 33 s) was used. Brain segmentation utilized 3D MPRAGE (TR = 1700 ms, TE = 2.21 ms, TI = 880 ms, flip angle 10°, FOV 256 × 232 × 176 mm^3^, resolution 1.0 × 1.0 × 1.0 mm^3^, scan time 3 min 37 s).

### 2.2 Relaxation maps reconstruction

R_2_ maps were reconstructed by Bloch modeling the dual‐echo TSE decay with measured flip angles. Specifically, R_2_ maps were reconstructed by a dictionary fitting method that accounts for the exact dual‐echo TSE pulse sequence, modeled by Bloch equations based on the actual RF pulse shapes and gradient waveforms and accounting for B_1_^+^ variations. This approach has been validated in human brain against a 32‐echo MESE sequence for mono‐exponential fitting.^40^ The actual applied flip angles in each voxel were determined by scaling the nominal flip angles with the acquired B_1_^+^ map. The dictionary considered T_2_ range from 0.01 to 2 s, in steps of 0.1 ms, and normalized B_1_^+^ range from 0.4 to 1.6, in steps of 0.005. $R_{2}^{*}$ maps were acquired by a mono‐exponential fitting of the MEGE magnitude with ARLO.^44^ $R_{2}^{\prime }$ maps were calculated by subtracting R_2_ from $R_{2}^{*}$ after interpolation of R_2_ maps to the same $R_{2}^{*}$ resolution and registering R_2_ maps to MEGE space using FSL.^45^ Negative $R_{2}^{\prime }$ values were set to zero since such values are not realistic.

### 2.3 Regions of interest segmentation

Four deep gray matter (DGM) structures (caudate, globus pallidum, putamen, and thalamus) were segmented in MPRAGE space using vol2Brain^46^ and three WM regions of interest (ROIs) (splenium, body and genu of corpus callosum) were segmented by registering the Johns Hopkins University White‐matter Atlas to MPRAGE space. Then, all ROIs were registered to MEGE space with FSL.^45^

### 2.4 Susceptibility Source Separation pipelines

Two susceptibility source separation methods were examined, χ‐separation^23^ and χ‐sepnet,^41^ which uses a deep learning approach. The reconstruction codes for both methods were downloaded from the chi‐separation toolbox (https://github.com/SNU‐LIST/chi‐separation) on 7 June 2024. Both methods determine independent paramagnetic and diamagnetic maps by linking $R_{2}^{\prime }$ to total susceptibility through the calculation of a relaxometric constant (D_r_), which is the slope of the linear regression between $R_{2}^{\prime }$ and absolute susceptibility. χ‐separation models the effect of susceptibility sources on the complex MRI signal as:

$$\begin{array}{cc} R_{2}^{\prime }\left(r\right)+i2\pi \cdot ∆f\left(r\right) & =\bar{D_{r,\text{para}}}\cdot \left|\chi_{\text{para}}\left(r\right)\right|+\bar{D_{r,\mathrm{dia}}}\cdot \left|\chi_{\mathrm{dia}}\left(r\right)\right|\\ & \;+i2\pi D_{f}\left(r\right)*\left(\chi_{\text{para}}\left(r\right)+\chi_{\mathrm{dia}}\left(r\right)\right) \end{array}$$

where r is position vector of a voxel, ∆f is frequency shift, $\bar{D_{r,\text{para}}}$ and $\bar{D_{r,\mathrm{dia}}}$ are nominal D_r_ for para and diamagnetic susceptibility sources, respectively, $\chi_{\text{para}}\left(r\right)$ and $\chi_{\mathrm{dia}}\left(r\right)$ are para and diamagnetic susceptibility within a voxel, respectively, $D_{f}$ is field perturbation kernel, and * represents a convolution. This equation was then written as a minimization problem with additional regularization terms and solved iteratively for $\chi_{\text{para}}\left(r\right)$ and $\chi_{\mathrm{dia}}\left(r\right)$ with a conjugate gradient descent algorithm (see Shin et al.^23^ for details). For χ‐sepnet, a 3D U‐net was trained with labels from χ‐separation using multi‐orientation head data. To date, a single D_r_ is calculated from paramagnetic regions values, and it is used for both the paramagnetic and diamagnetic susceptibility components.

The inputs for both methods were $R_{2}^{\prime }$ (calculated with the reconstructed $R_{2}^{*}$ and R_2_ maps); D_r_; data from the MEGE scan, which included the tissue field map, brain mask, and imaging frequency and B0 direction parameters. χ‐separation also required magnitude and noise maps from MEGE as inputs, as well as a QSM for an initial guess of the total susceptibility. The tissue field map was obtained by first unwrapping the phase of all echoes and estimating a field map using ROMEO,^47^ and then removing background field contributions using VSHARP^48^ with 12 mm kernel radius. The initial net susceptibility was reconstructed from the tissue field map by dipole field inversion with MEDI algorithm,^49^ which implicitly uses the brain average as a reference, and no further referencing was used.

The D_r_ was obtained in two ways: (1) using the default value from the source separation program^23^ (137 Hz/ppm) for all subjects or, (2) by calculating the D_r_ for each subject via a linear regression between the values of QSM and $R_{2}^{\prime }$ within iron‐rich DGM structures (caudate, globus pallidum, and putamen). The right and left sides for each structure were measured separately, resulting in six different points in the linear regression model. In accordance with the current literature, the same value of D_r_ was used for paramagnetic and diamagnetic components.

Each of the susceptibility source separation methods was performed twice, with default D_r_ and with subject‐estimated calculated D_r_, resulting in four different pipelines. The outputs for each susceptibility source separation pipeline include the paramagnetic and diamagnetic susceptibility maps. Both para and diamagnetic maps are always non‐negative, and the total χ map is calculated from the subtraction of the diamagnetic from the paramagnetic map.

### 2.5 Altered R_2_ maps as susceptibility source separation input

Errors in R_2_ were produced in one of three ways. First, by using a standard exponential fit of the dual‐echo TSE data, which is commonly used but known to substantially underestimate R_2_ values due to stimulated echo contamination, which depends upon the actual refocusing angles and the slice profile. Second, by using the Bloch‐modeled R_2_ maps with additional fabricated errors incorporated. The fabricated R_2_ errors used a global multiplication error, ranging from 75% to 125% of the original baseline R_2_ map in steps of 1%, generating 50 altered R_2_ maps with errors from −25% to 25% of the true value. Third, as well as the two direct R_2_ error approaches, an R_2_ approximation method was used based on past literature, where R_2_ is estimated from $R_{2}^{*}$. This method, as proposed by Dimov et al.,^25^ was previously applied to source separation for cases without R_2_ availability. It estimates the $R_{2}^{\prime }$ from $R_{2}^{*}$ by multiplying the $R_{2}^{*}$ values by 0.52, which is equivalent to estimating R_2_ as $R_{2}^{*}$ multiplied by 0.48.

The resultant altered R_2_ maps from all three approaches were then used to calculate new altered $R_{2}^{\prime }$ maps. These $R_{2}^{\prime }$ maps were used in the source separation pipelines to produce altered outputs of para and diamagnetic susceptibility maps. When not using the default D_r_ value, the D_r_ was also recalculated for each new $R_{2}^{\prime }$ map. In order to evaluate the influence of R_2_ on $R_{2}^{\prime }$, the ratio of R_2_ to $R_{2}^{*}$ was also calculated.

### 2.6 Susceptibility source separation error analysis

The resultant $R_{2}^{\prime }$ maps and the altered source separation program outputs, paramagnetic and diamagnetic susceptibility maps, were compared to the original baseline maps using difference maps and mean percentage error (MPE) metrics. Difference maps were produced by subtracting, for each voxel, the baseline map from the altered one. MPE was calculated, within each ROI, as 100 times the mean difference between altered minus baseline, divided by the mean of baseline map. Human brain ROI analysis included the aforementioned DGM and WM ROIs. The mean MPE for the 11 subjects was plotted against the range of R_2_ errors.

To account for possible CSF contamination in the automated segmentation, voxels were excluded when R_2_ <2.5 Hz (T_2_ >400 ms) or $R_{2}^{*}$ < 5 Hz ($T_{2}^{*}$ >200 ms), which might suggest partial CSF voluming. To evaluate if the altered maps differ from the baseline, paired t‐tests (α = 0.05) were performed with the distribution of 11 subjects mean values within each ROI, with the null hypothesis that difference between the samples from altered and baseline pipelines has a mean equivalent to zero. All processing (except for registration and segmentation) was done using MATLAB (version R2023a; MathWorks, MA, USA).

## 3 RESULTS

For the baseline map, the whole brain (including CSF) R_2_ mean value for all subjects was 11.7 Hz. In comparison, for the exponential‐fit and the $R_{2}^{*}$‐based estimated maps, whole brain R_2_ values were 6.7 Hz (−45% error) and 10.5 Hz (−18% error), respectively. For $R_{2}^{\prime }$, the mean values were 12.0 Hz and 11.5 Hz for the respective altered maps, compared to 7.2 Hz for baseline. For all subjects, the mean calculated D_r_ for baseline R_2_ was 142 ± 24 Hz/ppm (Max: 178 Hz/ppm, Min: 106 Hz/ppm), which differed from the default D_r_ of 137 Hz/ppm. No correlations between D_r_ and sex or age were found. The addition of R_2_ errors altered the D_r_s; considering the mean within subjects, exponential fit, $R_{2}^{*}$‐based estimation, −25% and 25% R_2_ error approaches produced a mean D_r_ of 164 Hz/ppm, 109 Hz/ppm, 159 Hz/ppm, and 121 Hz/ppm, respectively. Representing a mean change of 15%, −23%, 12% and −15% in D_r_ compared to the baseline mean value.

**FIGURE 1:**
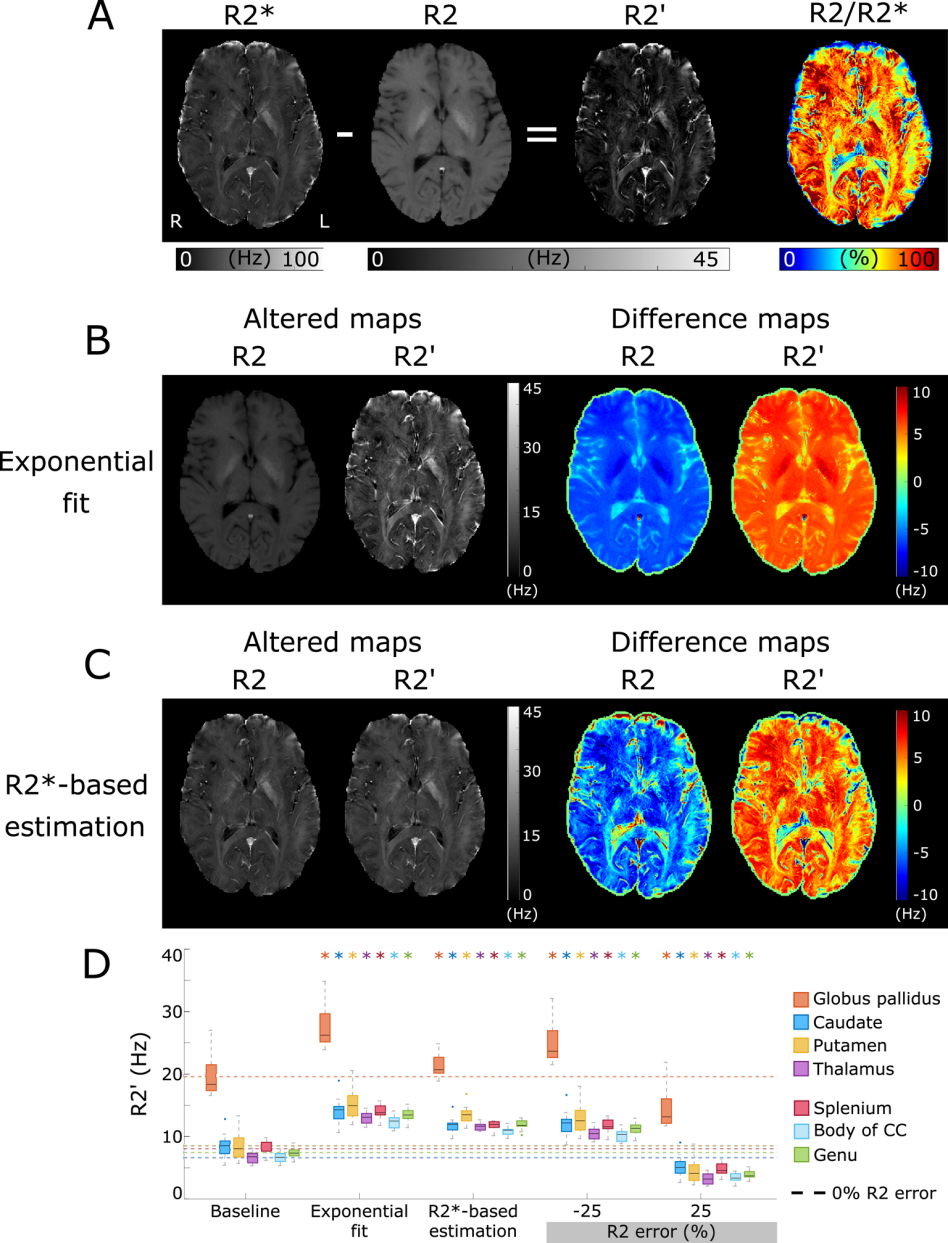
(A–C) Transverse relaxation and difference maps from a 26‐y‐old male subject. (A) Unaltered transverse relaxation maps showing the relationship between R_2_ and $R_{2}^{*}$ for producing $R_{2}^{\prime }$ and an R_2_/$R_{2}^{*}$ ratio map illustrating the influence of R_2_ in $R_{2}^{\prime }$. The R_2_/$R_{2}^{*}$ ratio has values of 60% or higher over most of the brain. (B) R_2_ and $R_{2}^{\prime }$ maps with R_2_ calculated by exponential fit, and (C) R_2_ and $R_{2}^{\prime }$ maps estimated from only $R_{2}^{*}$, assuming $R_{2}^{\prime }$ =0.52 $R_{2}^{*}$. The difference maps were calculated as the altered map minus the unaltered map. Both exponential fitting and $R_{2}^{*}$‐based estimations cause R_2_ underestimation that overestimates $R_{2}^{\prime }$. (D) Boxplots of $R_{2}^{\prime }$ mean values from all 11 subjects within GM and WM regions for baseline and four altered $R_{2}^{\prime }$ maps (exponential‐fit, $R_{2}^{*}$‐based estimation and ± 25% R_2_ error). The ground truth (baseline) result is also shown with its mean extended in a horizontal dashed color‐coded line. Outlier subjects are shown as dots. All distributions were significantly different (*p* < 0.05) from the baseline (as identified by the asterisks color‐coded by ROI).

Figure 1 illustrates the relationship between R_2_ and $R_{2}^{\prime }$ values and errors. The R_2_/$R_{2}^{*}$ ratio has values of 60% or higher in most regions (Figure 1A) and hence inaccurate R_2_ will translate into substantial $R_{2}^{\prime }$ errors. With exponential‐fit (Figure 1B), R_2_ is underestimated as stimulated echoes are not considered, yielding an overestimation in $R_{2}^{\prime }$. The degree of R_2_ underestimation with simple exponential fitting is dependent on the effective refocusing flip angles which varies with the B_1_^+^ distribution; thus, the exponential fit error has a spatial dependence. The $R_{2}^{*}$‐based estimation approach also resulted in an overestimation of $R_{2}^{\prime }$, and consequently, R_2_ underestimation (Figure 1C). Figure 1D shows regional $R_{2}^{\prime }$ boxplots for all R_2_ alteration approaches compared to baseline $R_{2}^{\prime }$ values. All of the alteration approaches resulted in $R_{2}^{\prime }$ values significantly different from the baseline within all evaluated ROIs.

**FIGURE 2:**
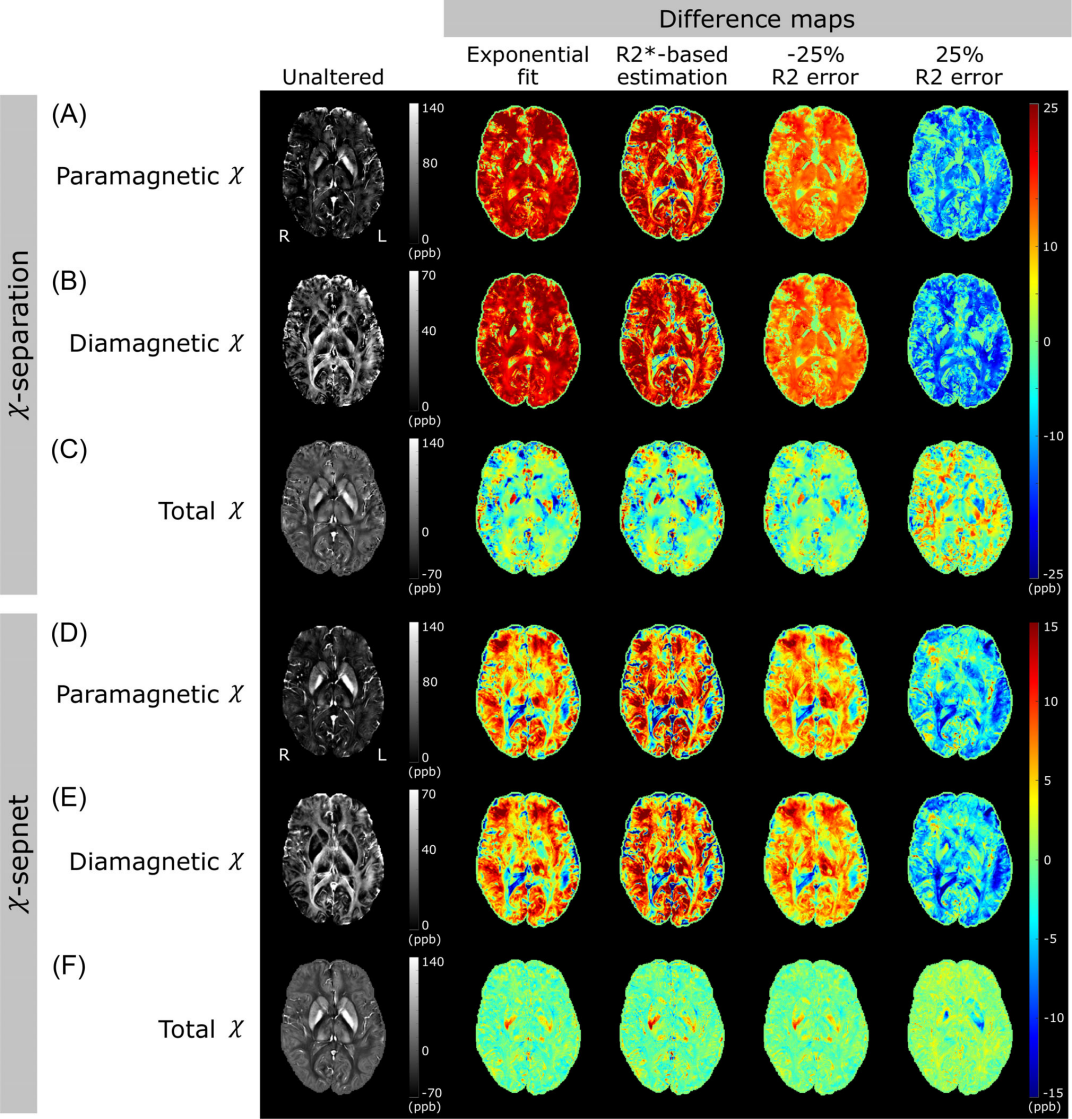
Susceptibility maps from a 26‐y‐old female subject with χ‐separation (A–C) and χ‐sepnet (D–F) from the unaltered R_2_ pipeline (left) and difference maps from altered pipelines (center and right) using the default relaxometric constant (D_r_). Unaltered total χ maps were acquired by subtracting the diamagnetic χ map from the paramagnetic one. The difference maps were calculated as the altered map minus the unaltered map. Due to reduced errors, the χ‐sepnet color range is from ±15% as opposed to ±25% in χ‐separation.

Para and diamagnetic difference maps for χ‐separation and χ‐sepnet outputs when using default D_r_ showed substantial errors (Figure 2). The χ‐separation method has higher errors in comparison to χ‐sepnet, with both methods showing regional variations in errors. The reduced $R_{2}^{\prime }$ error sensitivity of χ‐sepnet may arise from reduced reliance on $R_{2}^{\prime }$ in the model, as is also evident from the unaltered pipeline results that more effectively reduced the $R_{2}^{\prime }$ artifacts from air‐tissue interfaces in the frontal lobe. For both source separation methods, total χ maps (Figure 2C,F) were minimally affected by $R_{2}^{\prime }$ errors throughout the brain. Para and diamagnetic error maps follow the under or overestimation $R_{2}^{\prime }$ trend, as seen in the underestimated maps acquired with the 25% R_2_ error approach, while the other approaches resulted in overestimation. Exponential‐fit had similar results to $R_{2}^{*}$‐based estimation, with the latter having more error across the brain with χ‐sepnet and underestimation errors in the frontal lobe. For the case of calculated D_r_, similar results were observed, however, with slightly less error in most maps (except for the maps acquired with the $R_{2}^{*}$‐based estimation), compared to the default D_r_ approach (see Figure S1).

**FIGURE 3:**
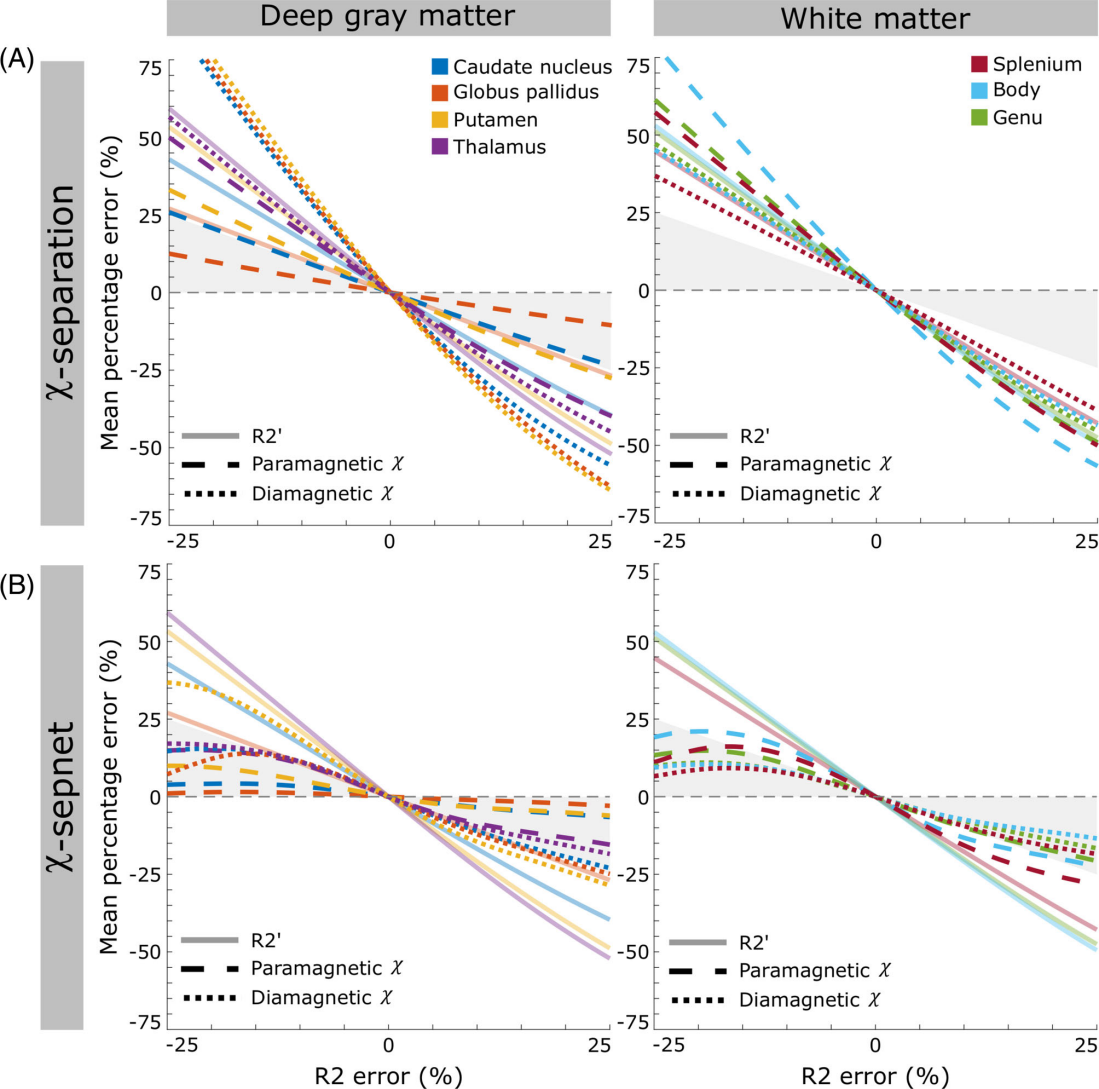
Mean MPE of $R_{2}^{\prime }$ and para and diamagnetic susceptibility maps versus R_2_ error for the 11 subjects. The χ‐separation (A) and χ‐sepnet (B) methods are shown within DGM (left column) and white matter (right column). The default relaxometric constant (D_r_) was used for both methods. Solid, dashed and dotted lines represent $R_{2}^{\prime }$, paramagnetic χ and diamagnetic χ, respectively. The shaded area represents an absolute output error less or equal to the absolute R_2_ input error. The solid $R_{2}^{\prime }$ lines are independent of the method used, enabling a reference for the relative errors between them.

To better understand the influence of R_2_ errors in para and diamagnetic susceptibility, MPE results from the incremental global R_2_ error variation for χ‐separation and χ‐sepnet using default D_r_ are shown in Figure 3. The effect of R_2_ error on $R_{2}^{\prime }$ MPE is highest in structures with lower $R_{2}^{*}$ and their relationship is linear, except when R_2_ overestimation introduced negative values of $R_{2}^{\prime }$ which were zeroed prior to the source separation algorithm. Effects of R_2_ error in susceptibility MPE were found to be inverse, as negative R_2_ errors introduce a positive MPE into susceptibility while positive R_2_ errors produces negative MPE. In the paramagnetic and diamagnetic χ maps with the χ‐separation method (Figure 3A), the regions with strong susceptibility sources aligning with the source separation map (i.e., iron‐rich GM for the paramagnetic χ map and myelin‐rich WM ROIs for the diamagnetic χ map) showed a response similar to the $R_{2}^{\prime }$ MPE but not linear and with consistently smaller errors. As for the regions with reduced susceptibility sources, opposite to the map focus (i.e., WM for paramagnetic χ map and GM for diamagnetic χ map), the inputted errors generated much larger errors than $R_{2}^{\prime }$ in the output MPE. The resulting MPE of χ‐separation outputs for small R_2_ input errors, as ±10%, was at least ±15%, for most of the analyzed ROIs. And this metric reached values greater than 25% for values as small as ±13% R_2_ error in most regions.

For χ‐sepnet (Figure 3B), all the susceptibility curves had a smaller output MPE than the respective $R_{2}^{\prime }$ curve and most of the curves stayed close to or under ±25% output MPE even for R_2_ input error as high as ±25%, which resulted in values about ±20% MPE (except for the diamagnetic curve of putamen and the paramagnetic curves of splenium and body of CC). In comparison to χ‐separation, χ‐sepnet outputs exhibited reduced error for both DGM and WM ROIs. For values as small as ±10% R_2_ input error, most ROIs had MPE of about ±9% or less, reaching values in the range of 0% to ±3% MPE in the paramagnetic map of 3 DGM ROIs. For the case of calculated D_r_, similar trends were observed, however, with generally less MPE error in para and diamagnetic maps, compared to the default D_r_ approach (see Figure S2).

Mean values (in ppb) and MPE (in %) for para and diamagnetic χ maps comparing default and calculated D_r_ approaches are shown in Table 1 (DGM ROIs) and Table 2 (WM ROIs). With χ‐separation, the calculated D_r_ produces less error than the default D_r_ for exponential fit and −25% and 25% R_2_ error alteration methods, while for the $R_{2}^{*}$‐based estimation, default D_r_ produced less error than calculated D_r_. As for the influence of D_r_ in χ‐sepnet, the default or calculated method showed similar mean values, with less than 2 ppb difference for most ROIs, and less than 4 ppb overall. The absolute difference between altered and baseline maps reached values as high as 32 ppb (in putamen diamagnetic value, using χ‐separation with calculated D_r_ and $R_{2}^{*}$‐based estimation), with the second highest value being 25 ppb (in globus pallidus paramagnetic value, using χ‐separation with default D_r_ and exponential‐fit R_2_).

**TABLE 1:** Paramagnetic and diamagnetic susceptibility mean values (in ppb) and mean percentage error (MPE) from deep gray matter regions.

	Paramagnetic χ				Diamagnetic χ			
	χ−separation		χ−sepnet		χ−separation		χ−sepnet	
	Default D_r_	Calculated D_r_	Default D_r_	Calculated D_r_	Default D_r_	Calculated D_r_	Default D_r_	Calculated D_r_
Globus Pallidus								
Unaltered								
Mean value (ppb)	132.0 (15.8)	129.9 (11.2)	134.6 (11.2)	133.6 (11.1)	18.6 (8.2)	16.8 (7.4)	8.3 (3.7)	7.8 (3.1)
Exponential fit								
Mean value (ppb)	157.0 (16.3)*	141.9 (11.3)*	134.2 (11.2)	134.1 (11.0)	42.5 (10.5)*	27.3 (8.5)*	8.0 (3.4)	7.3 (2.9)
MPE (%)	19.1 (1.8)	9.3 (1.9)	−0.3 (0.9)	0.4 (2.6)	145.8 (46.9)	70.6 (26.2)	−2.4 (11.1)	−0.8 (31.9)
R_2_*−based estimation								
Mean value (ppb)	134.6 (11.6)	153.7 (12.1)*	131.7 (10.7)*	132.4 (10.6)	20.6 (5.7)	39.9 (8.6)*	5.3 (2.5)*	6.2 (2.9)*
MPE (%)	2.3 (3.6)	18.5 (4.0)	−2.1 (1.5)	−0.9 (2.1)	17.4 (18.5)	157.9 (62.7)	−36.1 (10.3)	−19.6 (20.1)
−25% R_2_ error								
Mean value (ppb)	148.4 (16.1)*	136.8 (11.3)*	136.0 (11.3)*	134.7 (10.5)	33.8 (9.9)*	22.7 (8.0)*	8.8 (3.4)	8.0 (3.1)
MPE (%)	12.5 (1.3)	5.4 (1.2)	1.1 (1.4)	0.8 (1.5)	92.2 (27.2)	39.3 (15.2)	7.2 (15.3)	5.5 (21.4)
25% R_2_ error								
Mean value (ppb)	118.2 (15.2)*	124.5 (10.9)*	130.6 (11.2)*	133.7 (10.7)	7.7 (6.2)*	11.7 (6.2)*	6.4 (3.5)*	8.1 (3.4)
MPE (%)	−10.6 (1.2)	−4.1 (0.9)	−2.9 (0.8)	0.1 (0.8)	−62.8 (11.2)	−32.5 (10.0)	−24.9 (7.4)	3.5 (9.5)
Caudate								
Unaltered								
Mean value (ppb)	48.9 (9.3)	48.2 (8.8)	51.3 (5.8)	51.1 (5.4)	14.6 (5.9)	14.5 (8.6)	13.8 (4.0)	13.6 (4.9)
Exponential fit								
Mean value (ppb)	68.2 (10.0)*	60.8 (8.4)*	52.9 (5.6)*	52.7 (5.6)*	34.1 (7.5)*	26.2 (10.1)*	15.4 (3.7)*	14.9 (3.4)*
MPE (%)	40.8 (7.6)	27.3 (8.0)	3.2 (3.0)	3.4 (4.8)	150.0 (48.5)	99.5 (42.0)	13.4 (10.1)	14.2 (19.3)
R_2_*−based estimation								
Mean value (ppb)	60.4 (7.2)*	71.0 (7.5)*	52.0 (5.2)	52.8 (4.8)*	25.7 (4.9)*	37.8 (10.2)*	14.0 (2.7)	15.2 (4.0)*
MPE (%)	25.3 (10.9)	49.8 (16.0)	1.7 (5.3)	3.6 (3.8)	90.8 (46.3)	207.8 (110.1)	4.8 (16.5)	15.8 (17.1)
−25% R_2_ error								
Mean value (ppb)	61.2 (9.6)*	55.8 (8.3)*	53.2 (5.7)*	52.8 (5.2)*	26.3 (7.1)*	21.0 (9.4)*	15.4 (3.0)*	14.8 (3.8)*
MPE (%)	25.9 (5.0)	16.7 (5.3)	3.9 (4.2)	3.5 (3.5)	89.1 (26.6)	54.4 (22.7)	14.7 (14.7)	12.4 (12.9)
25% R_2_ error								
Mean value (ppb)	37.6 (8.8)*	40.6 (9.2)*	48.0 (6.3)*	49.4 (6.5)	6.8 (3.7)*	9.4 (7.3)*	10.7 (3.6)*	12.2 (5.1)
MPE (%)	−23.6 (4.0)	−16.2 (4.5)	−6.6 (2.6)	−3.4 (3.7)	−55.8 (7.4)	−39.4 (11.2)	−23.0 (6.0)	−11.0 (8.9)
Putamen								
Unaltered								
Mean value (ppb)	46.7 (12.4)	46.2 (12.5)	54.6 (11.4)	54.2 (11.2)	17.1 (5.2)	16.5 (6.7)	12.4 (2.1)	12.1 (2.9)
Exponential fit								
Mean value (ppb)	69.3 (12.9)*	61.6 (11.9)*	58.7 (10.3)*	58.9 (10.3)*	41.1 (6.4)*	32.2 (8.0)*	16.4 (1.7)*	16.0 (1.7)*
MPE (%)	51.7 (14.2)	36.1 (13.6)	8.4 (5.1)	9.5 (5.9)	149.5 (33.6)	104.3 (27.9)	34.2 (17.0)	37.8 (24.3)
R_2_*−based estimation								
Mean value (ppb)	63.4 (9.9)*	75.8 (10.8)*	58.9 (9.7)*	59.2 (9.7)*	35.3 (3.9)*	48.4 (8.2)*	16.0 (1.7)*	16.7 (2.3)*
MPE (%)	39.6 (17.3)	70.1 (27.4)	8.8 (6.6)	10.2 (5.9)	116.4 (37.8)	214.9 (66.6)	31.6 (21.8)	43.1 (24.7)
−25% R_2_ error								
Mean value (ppb)	61.2 (12.8)*	55.9 (12.0)*	59.6 (10.3)*	58.3 (10.3)*	32.4 (6.1)*	26.2 (7.4)*	16.7 (1.5)*	15.4 (2.6)*
MPE (%)	33.1 (9.0)	22.7 (8.7)	10.0 (5.6)	8.4 (5.1)	95.3 (20.8)	64.1 (17.3)	36.8 (17.0)	30.2 (14.9)
25% R_2_ error								
Mean value (ppb)	34.5 (11.7)*	36.8 (12.5)*	51.3 (10.9)*	52.1 (11.3)*	6.4 (3.1)*	8.3 (5.0)*	8.9 (2.1)*	9.8 (2.8)*
MPE (%)	−27.6 (6.2)	−21.9 (6.5)	−6.1 (1.1)	−4.2 (2.6)	−63.9 (6.5)	−52.1 (8.2)	−28.6 (4.5)	−18.9 (5.9)
Thalamus								
Unaltered								
Mean value (ppb)	26.7 (4.1)	26.4 (4.6)	26.5 (2.4)	26.3 (2.2)	23.9 (3.5)	23.8 (6.4)	24.3 (2.1)	24.1 (3.0)
Exponential fit								
Mean value (ppb)	48.4 (4.1)*	41.4 (5.4)*	29.8 (2.0)*	31.7 (3.0)*	46.2 (3.7)*	39.2 (7.3)*	28.2 (1.5)*	29.5 (1.2)*
MPE (%)	82.9 (12.6)	58.2 (12.0)	12.8 (6.8)	20.8 (13.3)	94.7 (14.9)	68.4 (16.7)	16.5 (7.7)	24.0 (15.4)
R_2_*−based estimation								
Mean value (ppb)	43.5 (3.0)*	54.3 (5.0)*	33.6 (2.0)*	31.2 (2.1)*	41.3 (2.4)*	52.6 (7.2)*	31.4 (1.8)*	29.5 (1.4)*
MPE (%)	64.9 (15.6)	109.4 (27.4)	27.2 (8.0)	18.8 (9.0)	75.1 (18.3)	129.5 (37.2)	29.8 (7.9)	23.6 (13.1)
−25% R_2_ error								
Mean value (ppb)	39.8 (4.3)*	35.2 (4.8)*	30.3 (2.4)*	30.8 (2.7)*	37.2 (3.6)*	32.6 (6.6)*	28.3 (1.8)*	28.5 (2.2)*
MPE (%)	50.0 (7.4)	34.0 (7.4)	14.9 (9.6)	17.3 (8.8)	56.6 (9.2)	39.4 (10.6)	17.1 (9.8)	18.9 (9.9)
25% R_2_ error								
Mean value (ppb)	16.2 (3.6)*	18.2 (4.2)*	22.4 (2.4)*	23.1 (2.1)*	13.3 (3.3)*	15.6 (6.0)*	19.8 (2.2)*	20.7 (2.9)*
MPE (%)	−39.9 (4.4)	−31.7 (5.5)	−15.4 (3.7)	−12.0 (4.6)	−45.0 (7.0)	−35.9 (8.8)	−18.4 (4.3)	−14.1 (4.5)

*Note*: Para and diamagnetic maps were acquired using χ−separation and χ−sepnet methods with default and calculated relaxometric constant. Values inside parentheses are the respective standard deviations. Asterisks indicate values significantly different (p < 0.05) from the baseline based on the performed paired t−test.

**TABLE 2:** Paramagnetic and diamagnetic susceptibility mean values (in ppb) and mean percentage error (MPE) for white matter regions.

	Paramagnetic χ				Diamagnetic χ			
	χ−separation		χ−sepnet		χ−separation		χ−sepnet	
	Default D_r_	Calculated D_r_	Default D_r_	Calculated D_r_	Default D_r_	Calculated D_r_	Default D_r_	Calculated D_r_
Splenium								
Unaltered								
Mean value (ppb)	23.5 (4.9)	23.5 (8.9)	20.9 (2.8)	20.0 (4.1)	35.9 (3.4)	35.8 (7.1)	37.3 (2.0)	36.3 (3.7)
Exponential fit								
Mean value (ppb)	44.8 (5.0)*	37.4 (9.4)*	20.6 (1.5)	22.4 (1.4)	57.4 (3.9)*	50.1 (8.1)*	37.5 (1.7)	39.2 (1.9)*
MPE (%)	93.9 (17.8)	65.7 (19.9)	−0.6 (12.1)	17.4 (29.1)	60.3 (5.4)	40.9 (6.1)	0.6 (6.2)	9.0 (13.7)
R_2_*−based estimation								
Mean value (ppb)	36.7 (3.2)*	48.7 (8.8)*	22.2 (1.3)	21.0 (1.3)	49.5 (2.7)*	60.6 (7.5)*	39.1 (1.8)*	37.7 (2.0)
MPE (%)	59.6 (18.8)	120.7 (42.2)	7.6 (12.5)	9.4 (24.6)	38.4 (8.3)	71.7 (16.1)	4.9 (6.2)	4.7 (12.7)
−25% R_2_ error								
Mean value (ppb)	36.5 (4.8)*	31.4 (8.8)*	22.8 (1.6)	23.3 (2.3)*	49.1 (3.7)*	44.1 (7.5)*	39.7 (2.8)*	39.9 (2.6)*
MPE (%)	57.3 (11.1)	37.8 (12.4)	11.1 (17.8)	20.2 (19.5)	36.9 (3.4)	23.9 (4.4)	6.6 (8.7)	10.6 (9.6)
25% R_2_ error								
Mean value (ppb)	12.1 (4.5)*	15.0 (8.2)*	15.0 (2.5)*	16.4 (3.9)*	22.1 (3.4)*	25.3 (6.8)*	30.4 (1.3)*	31.9 (3.0)*
MPE (%)	−50.0 (8.1)	−38.9 (9.4)	−28.4 (4.4)	−18.0 (6.6)	−38.8 (4.1)	−30.1 (5.7)	−18.5 (2.3)	−12.1 (3.8)
Body of CC								
Unaltered								
Mean value (ppb)	15.5 (3.4)	15.5 (5.2)	16.8 (2.3)	16.4 (2.7)	30.6 (3.2)	30.2 (6.4)	33.8 (1.7)	33.3 (3.2)
Exponential fit								
Mean value (ppb)	35.4 (4.4)*	29.3 (6.3)*	18.1 (1.1)*	18.9 (1.1)*	52.6 (3.7)*	45.5 (7.3)*	35.4 (2.2)*	36.2 (2.2)*
MPE (%)	132.8 (25.3)	94.9 (24.8)	9.1 (12.5)	18.5 (23.3)	72.9 (7.6)	52.0 (9.0)	5.0 (5.9)	9.6 (11.0)
R_2_*−based estimation								
Mean value (ppb)	29.5 (3.5)*	40.1 (6.7)*	19.7 (0.8)*	19.6 (1.0)*	46.8 (2.6)*	57.7 (6.9)*	37.1 (2.1)*	36.7 (2.3)*
MPE (%)	94.7 (26.9)	172.2 (55.7)	18.9 (14.1)	22.0 (18.0)	53.9 (9.9)	94.7 (22.6)	9.9 (6.3)	10.6 (8.9)
−25% R_2_ error								
Mean value (ppb)	27.4 (4.2)*	23.5 (5.7)*	19.6 (1.1)*	19.6 (1.9)*	44.3 (3.6)*	39.3 (6.8)*	36.9 (3.1)*	36.8 (2.7)*
MPE (%)	79.2 (14.4)	55.0 (15.5)	19.1 (20.7)	21.3 (16.8)	45.2 (4.6)	31.1 (6.0)	9.4 (9.5)	10.8 (8.1)
25% R_2_ error								
Mean value (ppb)	6.8 (2.2)*	8.6 (4.3)*	13.0 (1.7)*	13.6 (2.1)*	17.3 (2.6)*	20.0 (5.7)*	29.2 (1.5)*	29.8 (2.0)*
MPE (%)	−56.7 (6.3)	−46.7 (8.9)	−22.6 (4.6)	−17.0 (8.1)	−43.5 (3.6)	−34.7 (5.4)	−13.5 (2.5)	−10.2 (4.1)
Genu								
Unaltered								
Mean value (ppb)	22.5 (4.3)	22.2 (5.3)	21.4 (2.7)	21.5 (3.6)	30.3 (2.8)	30.2 (6.0)	32.9 (2.6)	33.0 (3.6)
Exponential fit								
Mean value (ppb)	44.0 (5.1)*	36.8 (6.6)*	22.6 (1.6)	22.8 (1.7)	52.8 (3.3)*	45.8 (7.3)*	34.7 (2.9)*	34.9 (2.9)*
MPE (%)	98.0 (16.9)	68.1 (13.9)	6.4 (9.6)	8.2 (16.1)	74.3 (5.8)	52.9 (7.0)	5.5 (5.6)	6.7 (10.2)
R_2_*−based estimation								
Mean value (ppb)	38.3 (4.4)*	50.1 (7.9)*	22.6 (1.8)*	23.0 (2.0)*	46.8 (2.5)*	58.3 (7.7)*	34.8 (2.9)*	35.1 (2.9)*
MPE (%)	72.5 (17.2)	131.2 (31.6)	6.3 (8.4)	8.8 (12.7)	54.8 (8.0)	95.9 (18.9)	6.0 (4.9)	7.0 (8.1)
−25% R_2_ error								
Mean value (ppb)	36.0 (5.0)*	31.0 (6.1)*	24.0 (1.9)*	23.5 (2.3)*	44.6 (3.2)*	39.8 (6.7)*	36.1 (3.1)*	35.6 (3.1)*
MPE (%)	61.3 (9.9)	41.0 (8.5)	13.3 (12.5)	11.1 (10.0)	47.2 (3.9)	32.8 (5.3)	9.9 (7.3)	8.4 (6.6)
25% R_2_ error								
Mean value (ppb)	11.6 (3.1)*	13.8 (4.4)*	16.9 (2.0)*	18.2 (2.9)*	16.7 (2.8)*	19.0 (5.3)*	27.5 (3.1)*	28.8 (3.1)*
MPE (%)	−49.1 (5.9)	−38.9 (6.7)	−20.7 (5.0)	−14.9 (6.4)	−45.4 (4.6)	−37.8 (5.9)	−16.6 (4.2)	−12.4 (4.6)

*Note*: Para and diamagnetic maps were acquired using χ−separation and χ−sepnet methods with default and calculated relaxometric constant. Values inside parentheses are the respective standard deviations. Asterisks indicate values significantly different (p < 0.05) from the baseline based on the performed paired t−test.

*Abbreviations*: CC, Corpus callosum.

Comparing altered paramagnetic and diamagnetic χ maps from the same pipeline, the absolute errors were similar in ppb, with the highest variation in these differences being 4.6 ppb in the corpus callosum when using fixed D_r_ with χ‐separation and 25% R_2_ error input (in this case, the error was −8.7 ppb for paramagnetic χ map, and −13.3 ppb for diamagnetic χ map). χ‐sepnet showed lower variance between these differences than χ‐separation, with the highest variation seen being 2.0 ppb in the globus pallidus when using fixed D_r_ and 25% R_2_ error input, while all the other pipelines (χ‐sepnet for other ROIs, D_r_ approach or input R_2_ altered map) had a variation of 1.0 ppb or less. Overall, χ‐sepnet showed less sensitivity to R_2_ errors and reduced variance among subjects; for most of the analyzed alteration methods, the values within all analyzed ROIs remain close to the mean value of the baseline maps, suggesting that χ‐sepnet is less dependent on $R_{2}^{\prime }$ values.

## 4 DISCUSSION AND CONCLUSIONS

This work evaluated the role of R_2_ mapping accuracy in susceptibility source separation for human brain. The production of R_2_ errors was done in three ways: the commonly performed simple exponential fit, a $R_{2}^{*}$‐based approximation, or percent error adjustment of each voxel. The latter approach has a global error in R_2_ estimation, while the two former two use more local errors. Errors in R_2_ translated into errors in $R_{2}^{\prime }$ that altered the susceptibility source separation outputs.

We found that these errors in R_2_ can cause substantial deviations in the resultant paramagnetic and diamagnetic maps, reaching as high as twice the percentage R_2_ error, depending on the ROI, D_r_ approach and/or the source separation method. For instance, with ±25% R_2_ error input the output susceptibility maps had a mean difference to baseline (across subjects and ROIs) of 12 ppb (49% error) and 8 ppb (34% error) for χ‐separation with default and calculated D_r_, respectively, and 3.5 ppb (15% error) and 2.6 ppb (12% error) for χ‐sepnet with default and calculated D_r_, respectively. R_2_ error with exponential fitting can exceed −25%; thus, larger errors occur in separated maps. For the $R_{2}^{*}$‐based approximation, the mean R_2_ error was −18% across the whole brain, while the mean difference from baseline susceptibility component outputs were 13 ppb (57% error) and 26 ppb (117% error) for χ‐separation with default and calculated D_r_, respectively, and 2 ppb (8% error) and 2 ppb (11% error) for χ‐sepnet with default and calculated D_r_, respectively. High MPE were observed in areas where the baseline susceptibility component values were small (for example, DGM in the diamagnetic χ map), where a few ppb error represents a substantial percentage change.

The sum of paramagnetic and diamagnetic absolute maps is highly affected by R_2_ errors, due to their dependence on $R_{2}^{\prime }$: $R2^{\prime }=D_{r}\cdot \left(\left|\chi_{\text{para}}\right|+\left|\chi_{\mathrm{dia}}\right|\right)$. In contrast, the difference between these absolute maps, which is the total susceptibility: $\chi_{\text{total}}=\left|\chi_{\text{para}}\right|-\left|\chi_{\mathrm{dia}}\right|$; is in essence independent of $R_{2}^{\prime }$, thus stays stable with R_2_ errors. Therefore, an increase in the sum mandated by an R_2_ error might require a disproportionately larger change in $\chi_{\text{para}}$ and $\chi_{\mathrm{dia}}$ to maintain the same difference (i.e., $\chi_{\text{total}}$).

χ‐sepnet was less sensitive to R_2_ errors and D_r_ value and showed less variance between subjects. From our results, the algorithm for χ‐sepnet may tend to underweight unexpected $R_{2}^{\prime }$ values, leading to less sensitivity to its errors. Indeed, the χ‐sepnet algorithm underweights $R_{2}^{\prime }$ in comparison to χ‐separation, as it relies on $R_{2}^{\prime }$, tissue field map, and QSM in its loss cost function,^28^ compared to only the former two ($R_{2}^{\prime }$ and tissue field map) in χ‐separation.^23^ Moreover, χ‐sepnet tended to have outputs within a limited ppb range, potentially based on the range of values used for training the model. This behavior might help in correcting the unexpected values from poorly fitted voxels; but it could also mean that the χ‐sepnet model may not properly deal with input values outside of the training range. Thus, while χ‐sepnet was more robust to $R_{2}^{\prime }$ errors than χ‐separation, this finding does not answer the question as to which is more accurate. Past work on comparing χ‐sepnet to χ‐separation (with multi‐orientation head data), found that the deep‐learning based model yields more smooth results, due to its denoising nature.^50^ A previous repeatability study^51^ also showed that χ‐sepnet had better reliability than χ‐separation, which can be related to the reduced sensitivity of χ‐sepnet to $R_{2}^{\prime }$ errors.

Using the default D_r_ resulted in higher percentage errors than when D_r_ was calculated individually for most of the R_2_ alteration approaches analyzed, with both susceptibility source separation methods. Since the paramagnetic and diamagnetic susceptibilities are dependent on the ratio of $R_{2}^{\prime }$/D_r_, errors in $R_{2}^{\prime }$ may be minimized by new D_r_ calculations. For example, erroneously increased $R_{2}^{\prime }$ values will yield an increased D_r_ calculation, preserving a $R_{2}^{\prime }$/D_r_ ratio somewhat close to the accurate. However, this is only applicable when the relationship between the ROIs utilized in the linear regression for the estimation of D_r_ is preserved (i.e., a global error affecting the slope of all values, as in the ±25% R_2_ error approaches). For local errors, as the $R_{2}^{*}$‐estimation, the ratio for each ROI might be different; thus, the newly estimated D_r_ could alter the $R_{2}^{\prime }$/D_r_ ratio. Additionally, in this work, potential errors in QSM were not considered which could also play a role in the estimation of D_r_ and altering $R_{2}^{\prime }$/D_r_ ratio.

For the dualecho TSE sequence, it is known that direct exponential fitting leads to inaccurate R_2_ maps.^40^ Even when using a large number of echoes in MESE sequences, and skipping the first echo, exponential fitting will still lead to R_2_ errors, which will tend to vary across the brain depending on the B_1_^+^ variation, yielding different degrees of stimulated echoes.^39^ For example, in the dual echo TSE approach used in this study, exponential errors were ≈45%, in average, across the whole brain leading to large errors in source separation outputs. Thus, simple exponential fitting for R_2_ should be avoided in susceptibility source separation studies.

The R_2_ alteration approaches analyzed in this work included cases of both under‐ and overestimation of R_2_. In practice, R_2_ underestimation is far more common, as seen in the exponential‐fit, and $R_{2}^{*}$‐based approaches. However, R_2_ overestimation might occur when the apparent R_2_ is measured with large interecho spacings, leading to more rapid decay from diffusion; inadequate slice gap or incidental magnetization transfer effects when using a short TR (as studied by Radunsky et al^52^); or for the $R_{2}^{*}$‐based estimate, R_2_ may be overestimated when the $R_{2}^{*}$ is overestimated such as near air‐tissue interfaces.

Three recent studies avoided R_2_ mapping by directly estimating $R_{2}^{\prime }$ from $R_{2}^{*}$; Dimov et al.^25^ and Kan et al.^27^ estimated $R_{2}^{\prime }$ as $R_{2}^{*}$ multiplied by a factor (calculated from a linear fit when both R_2_ and $R_{2}^{*}$ were available), while Kim et al.^28^ used a neural network (R_2_PRIMEnet) to estimate $R_{2}^{\prime }$ from $R_{2}^{*}$. These approaches are valuable to save the time required to acquire an accurate R_2_ map; however, any error introduced into $R_{2}^{\prime }$ will yield errors in the susceptibility source separation outputs, as shown here. We tested the $R_{2}^{*}$‐based estimation proposed by Dimov et al.^25^ which produced a mean of 18% output susceptibility component error for the evaluated pipelines.

More generally, most our work has focused on R_2_ errors, due to the greater difficulty in obtaining accurate R_2_ measurement than $R_{2}^{*}$. However, all the results presented lead to $R_{2}^{\prime }$ errors that could have arisen from R_2_ or $R_{2}^{*}$. Thus this work is generalizable to generic $R_{2}^{\prime }$ errors as well. By focusing on R_2_ errors, their effect on source separation outputs varies with the $R_{2}^{*}$ to R_2_ ratio, with less sensitivity to R_2_ errors in regions of greater $R_{2}^{*}$. These large $R_{2}^{*}$ values are typically in regions dominated by iron, which are less affected by R_2_ errors, at least for the paramagnetic component.

Limitations of this work include that only two source separation algorithms were evaluated. We chose to use two common approaches (one iterative and one deep learning) to illustrate effects of R_2_ errors. Another limitation is the use of a global R_2_ alteration factor, which does not account fully for local variations since each region is affected by the same percent change. In addition, two realistic and non‐global alteration approaches were also applied. A further limitation is that the baseline R_2_ map used a dual‐echo TSE sequence. The low number of echoes is sensitive to noise and limits the range of accurate R_2_ values given that the final echo TE is at 93 ms. A MESE sequence with many echoes is preferable but is not time efficient. Furthermore, the dual‐echo approach (acquired in 3 min) has been proven to give accurate results when Bloch modeling is used and a B_1_^+^ map provides the exact flip angles.^40^ Lastly, different spatial resolutions were used for the dual‐echo TSE and MEGE sequences, which may introduce registration errors in the obtained $R_{2}^{\prime }$ maps, although R_2_ maps were interpolated to the same resolution as $R_{2}^{*}$ maps.

In conclusion, paramagnetic and diamagnetic outputs of susceptibility source separation methods that rely on $R_{2}^{\prime }$ can be strongly biased by R_2_ errors that may occur with simple R_2_ fitting approaches or R_2_ approximations. R_2_ errors substantially affected the outputs of χ‐separation and χ‐sepnet in markedly different ways, with χ‐separation having larger errors, reaching up to twice the input R_2_ error, partly due to its stronger algorithmic weighting of the $R_{2}^{\prime }$ input. Therefore, proper R_2_ modeling that accounts for the B_1_^+^ variations and stimulated echoes should be considered, or, when R_2_ is not available, more advanced prediction methods may be needed.

## Supporting information


**Figure S1.** Susceptibility maps from a 26 year‐old female subject with χ‐separation (A‐C) and χ‐sepnet (D‐F) from the unaltered R_2_ pipeline (left) and difference maps from altered pipelines using the calculated relaxometric constant (D_r_). For this subject, the calculated D_r_ for unaltered pipeline was 118 Hz/ppm. Unaltered total χ maps were acquired by subtracting the diamagnetic χ map from the paramagnetic one. The difference maps were calculated as the altered map minus the unaltered map, for both methods. Due to reduced errors, the χ‐sepnet color range from ±15% as opposed to ±25% in χ‐separation.
**Figure S2.** Mean MPE of $R_{2}^{\prime }$ and para and diamagnetic susceptibility maps versus R_2_ error for the 11 subjects. The χ‐separation (A) and χ‐sepnet (B) methods are shown within deep gray matter (left column) and white matter (right column). Calculated relaxometric constant (D_r_) was used for both methods. Solid, dashed and dotted lines represent $R_{2}^{\prime }$, paramagnetic χ and diamagnetic χ, respectively. The shaded area represents an absolute output error less or equal to the absolute R_2_ input error. The solid $R_{2}^{\prime }$ lines are independent of the method used, enabling a reference for the relative errors between them.
