# Supplementary material for: Importance of R2 accuracy in susceptibility source separation
Source: Magn Reson Med. 2025 Aug 16;95(1):157–71. doi: 10.1002/mrm.70034 (PMC12620178; doi:10.1002/mrm.70034)
Supplement: Supplementary file 1 — Figure S1. Susceptibility maps from a 26 year‐old female subject with χ‐separation (A‐C) and χ‐sepnet (D‐F) from the unaltered R2 pipeline (left) and difference maps from altered pipelines using the calculated relaxometric constant (Dr). For this subject, the calculated Dr for unaltered pipeline was 118 Hz/ppm. Unaltered total χ maps were acquired by subtracting the diamagnetic χ map from the paramagnetic one. The difference maps were calculated as the altered map minus the unaltered map, for both methods. Due to reduced errors, the χ‐sepnet color range from ±15% as opposed to ±25% in χ‐separation. Figure S2. Mean MPE of R2′ and para and diamagnetic susceptibility maps versus R2 error for the 11 subjects. The χ‐separation (A) and χ‐sepnet (B) methods are shown within deep gray matter (left column) and white matter (right column). Calculated relaxometric constant (Dr) was used for both methods. Solid, dashed and dotted lines represent R2′, paramagnetic χ and diamagnetic χ, respectively. The shaded area represents an absolute output error less or equal to the absolute R2 input error. The solid R2′ lines are independent of the method used, enabling a reference for the relative errors between them. [file MRM-95-157-s001.docx]

**SUPPLEMENTARY FIGURES**


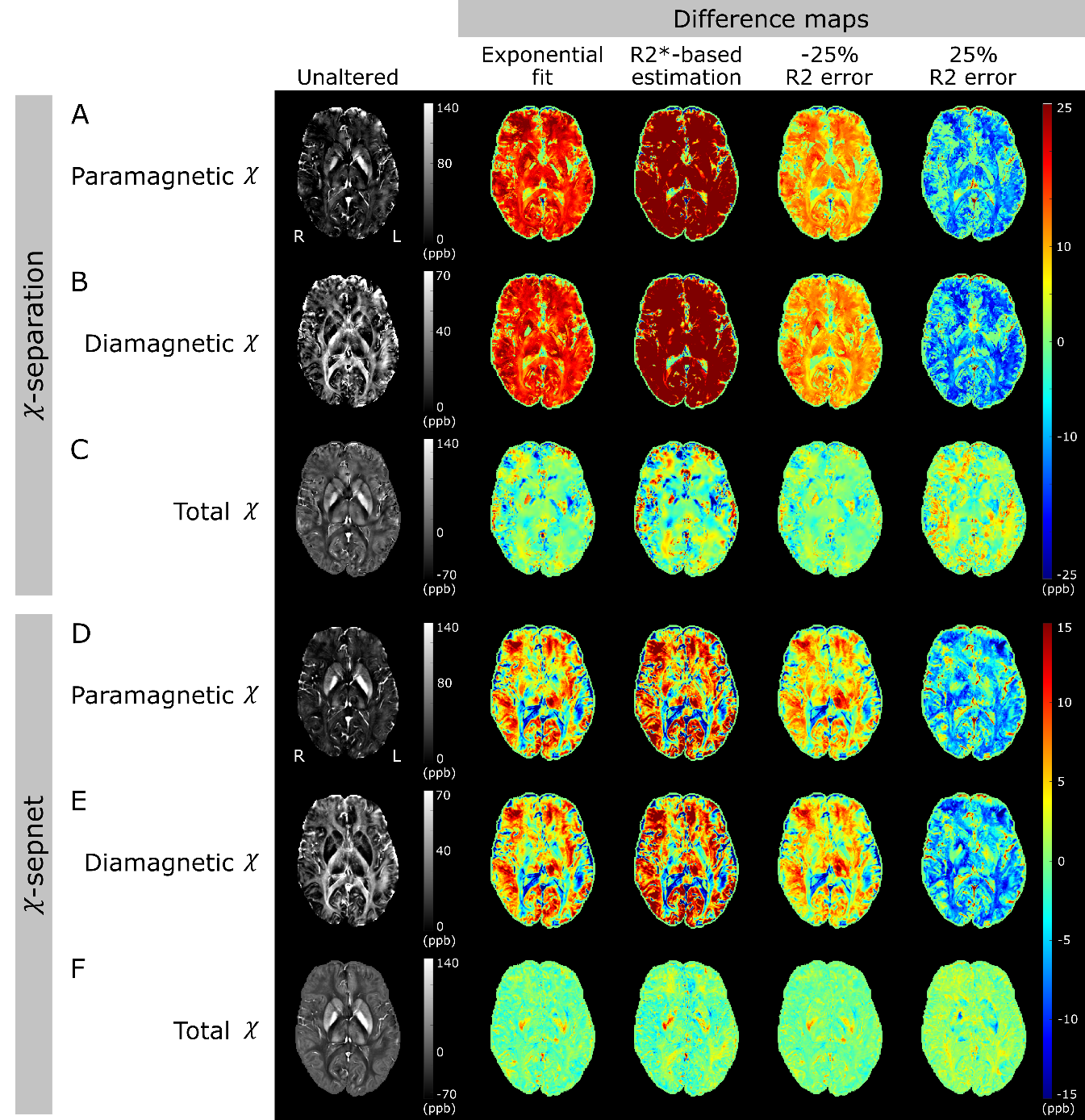


**Figure S1.** Susceptibility maps from a 26 year-old female subject with χ-separation (A-C) and χ-sepnet (D-F) from the unaltered R2 pipeline (left) and difference maps from altered pipelines using the calculated relaxometric constant (Dr). For this subject, the calculated Dr for unaltered pipeline was 118 Hz/ppm. Unaltered total χ maps were acquired by subtracting the diamagnetic χ map from the paramagnetic one. The difference maps were calculated as the altered map minus the unaltered map, for both methods. Due to reduced errors, the χ-sepnet color range from ±15% as opposed to ±25% in χ-separation.


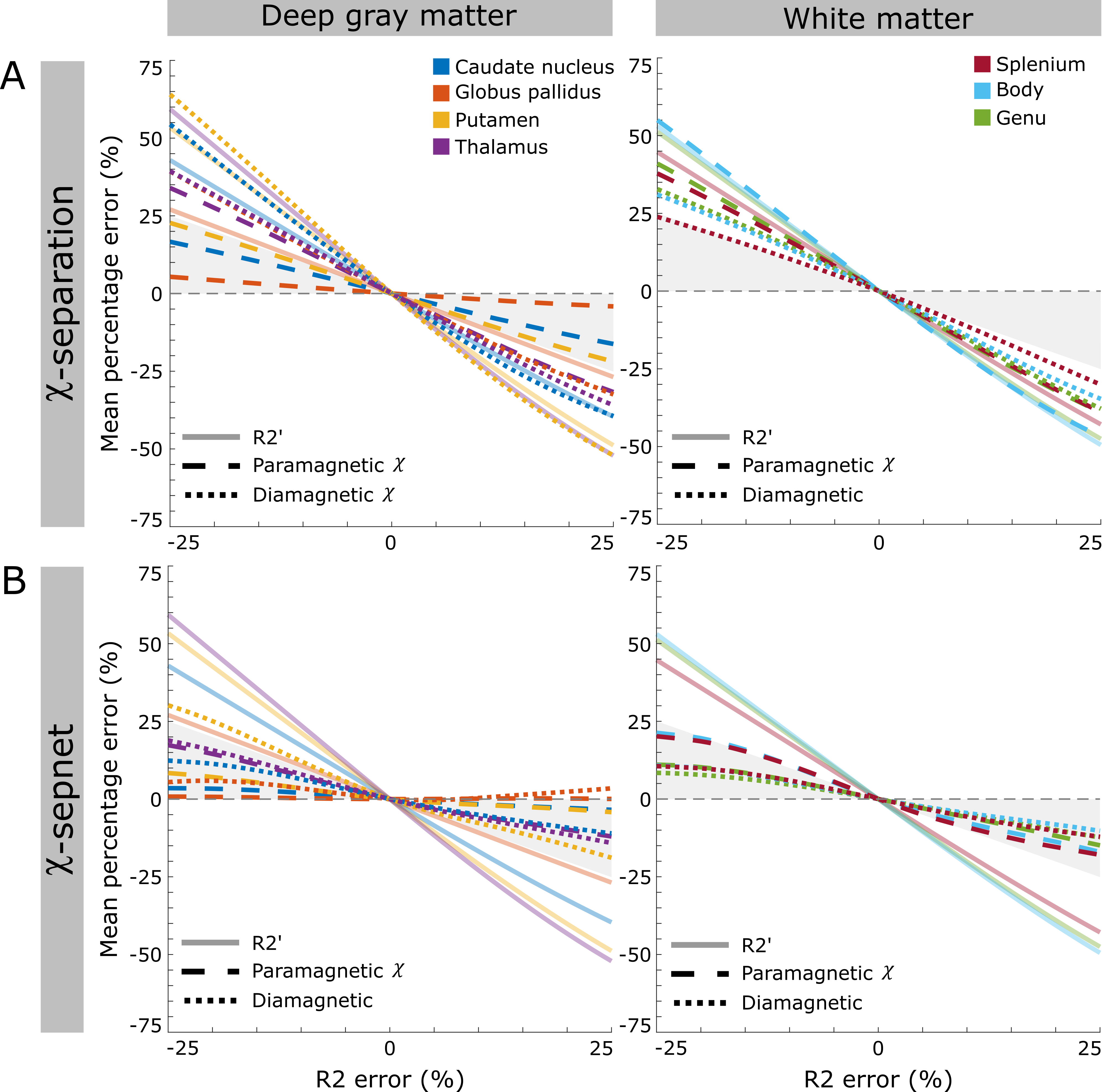


**Figure S2.** Mean MPE of R2’ and para and diamagnetic susceptibility maps versus R2 error for the 11 subjects. The χ-separation (A) and χ-sepnet (B) methods are shown within deep gray matter (left column) and white matter (right column). Calculated relaxometric constant (Dr) was used for both methods. Solid, dashed and dotted lines represent R2’, positive χ and negative χ, respectively. The shaded area represents an absolute output error less or equal to the absolute R2 input error. The solid R2’ lines are independent of the method used, enabling a reference for the relative errors between them.
